# Identification of key amino acid residues in AtUMAMIT29 for transport of glucosinolates

**DOI:** 10.3389/fpls.2023.1219783

**Published:** 2023-07-17

**Authors:** Lasse Meyer, Christoph Crocoll, Barbara Ann Halkier, Osman Asghar Mirza, Deyang Xu

**Affiliations:** ^1^ Department of Plant and Environmental Sciences, Faculty of Science, University of Copenhagen, Frederiksberg, Denmark; ^2^ Department of Drug Design and Pharmacology, Faculty of Health and Medical Sciences, University of Copenhagen, Copenhagen, Denmark

**Keywords:** structure/function, glucosinolate exporter, UMAMIT, key amino acid residues, structure prediction, substrate transporting cavity

## Abstract

Glucosinolates are key defense compounds of plants in Brassicales order, and their accumulation in seeds is essential for the protection of the next generation. Recently, members of the Usually Multiple Amino acids Move In and Out Transporter (UMAMIT) family were shown to be essential for facilitating transport of seed-bound glucosinolates from site of synthesis within the reproductive organ to seeds. Here, we set out to identify amino acid residues responsible for glucosinolate transport activity of the main seed glucosinolate exporter UMAMIT29 in *Arabidopsis thaliana*. Based on a predicted model of UMAMIT29, we propose that the substrate transporting cavity consists of 51 residues, of which four are highly conserved residues across all the analyzed homologs of UMAMIT29. A comparison of the putative substrate binding site of homologs within the brassicaceous-specific, glucosinolate-transporting clade with the non-brassicaceous-specific, non-glucosinolate-transporting UMAMIT32 clade identified 11 differentially conserved sites. When each of the 11 residues of UMAMIT29 was individually mutated into the corresponding residue in UMAMIT32, five mutant variants (UMAMIT29#V27F, UMAMIT29#M86V, UMAMIT29#L109V, UMAMIT29#Q263S, and UMAMIT29#T267Y) reduced glucosinolate transport activity over 75% compared to wild-type UMAMIT29. This suggests that these residues are key for UMAMIT29-mediated glucosinolate transport activity and thus potential targets for blocking the transport of glucosinolates to the seeds.

## 1 Introduction

Glucosinolates are amino-acid-derived, specialized metabolites characteristic of the Brassicales order. Glucosinolates accumulate to high levels in seeds with no *de novo* synthesis, thus relying on translocation from source tissue (Nour-Eldin and Halkier, 2009). Recently, members of the Usually Multiple Amino acids Move In and out Transporter (UMAMIT) family—UMAMIT29, UMAMIT30, and UMAMIT31—were identified as glucosinolate facilitators essential for exporting seed-bound glucosinolates from the site of synthesis within the reproductive organ to seeds in *Arabidopsis thaliana* (hereafter *Arabidopsis*) (Xu et al., 2023). UMAMIT29, UMAMIT-30, and UMAMIT31 facilitate glucosinolate efflux from biosynthetic cells with a uniport mechanism following the electrochemical gradient.

As the UMAMIT family name implies, a few of the characterized UMAMITs are bidirectional amino acid transporters containing 10 transmembrane-spanning helices (Müller et al., 2015; Besnard et al., 2016; Besnard et al., 2018; Zhao et al., 2021). In a recent study, a tertiary structure of UMAMIT14 was predicted to form homodimers via helix 5 and 10 based on homology modelling, and mutation of residues in these helices resulted in reduction of amino acid import activity (Zhao et al., 2021). However, both the substrate transporting cavity and the determinants of substrate specificity within the cavity of UMAMITs for any substrate remains unknown.

Here, we set out to identify the determinants for glucosinolate transport activity using UMAMIT29—the major UMAMIT with a role in glucosinolate seed accumulation—as case study. Based on distance-based protein folding tools, we predict the substrate-transporting cavity of UMAMIT29. By comparing differentially conserved residues within the predicted cavities between glucosinolate-transporting and non-glucosinolate-transporting UMAMITs, we identify 11 candidate residues. Transport activity assays of mutant variants with a single point amino acid substitution in each site UMAMIT29 suggest that five of these residues are critical for glucosinolate transport.

## 2 Results

### 2.1 Substrate transporting cavity of UMAMIT29

**Figure 1:**
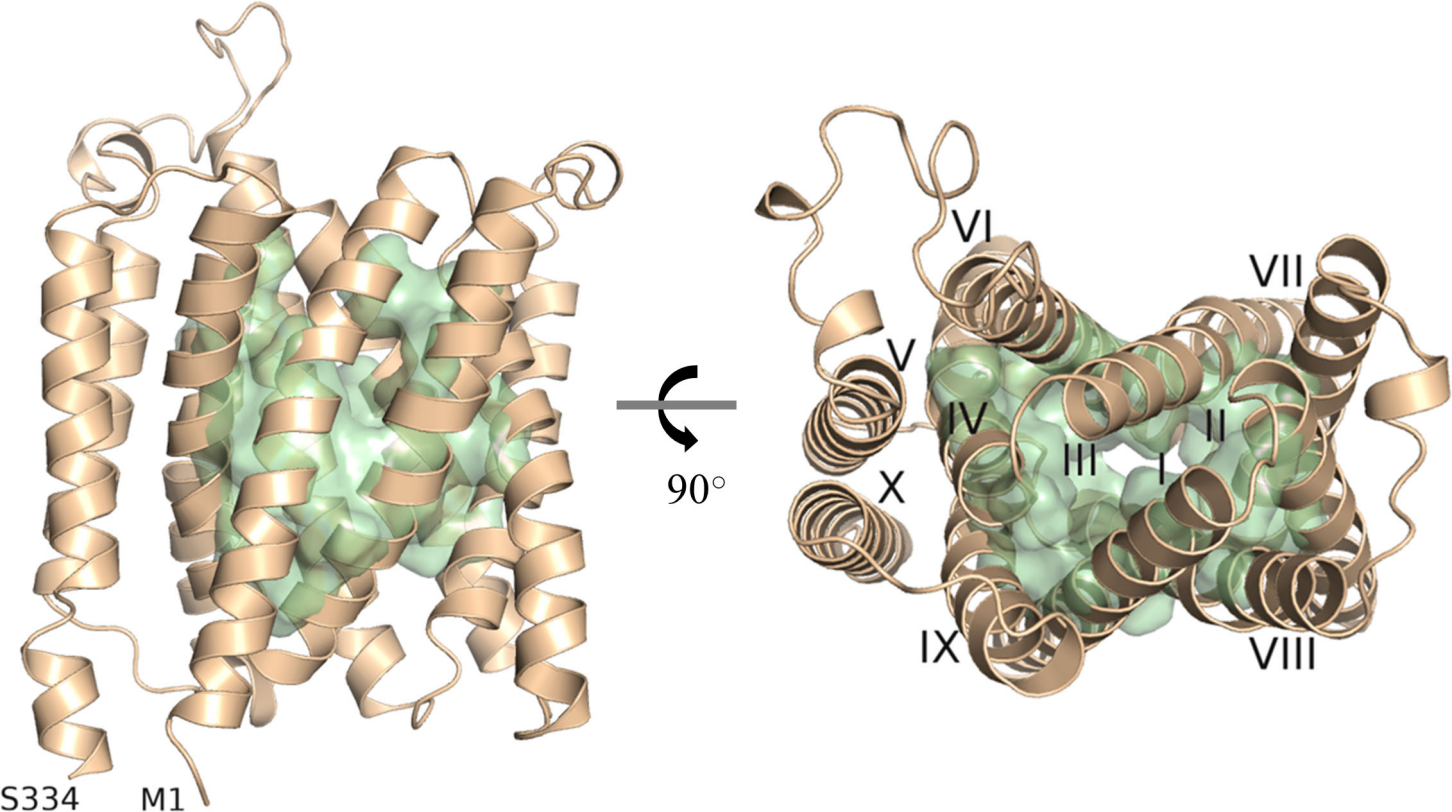
Depiction of the predicted substrate transporting cavity of UMAMIT29. A protein model of AtUMAMIT29 was generated, and 51 residues were predicted to constitute the transport cavity (green). The protein model is shown from the side (left panel) and from the top (right panel) where each of the helices are numbered (I–X). The protein model was generated using RaptorX and displayed in PyMOL 2.4.

As there is yet no experimentally determined structure available for any member of the UMAMIT family, we generated a model of UMAMIT29 in an apparent occluded conformation by the *ab initio* protein modelling tool RaptorX (Ma et al., 2015; Wang et al., 2016; Wang et al., 2017). Based on our model, we proposed a substrate-transporting cavity of UMAMIT29 defined by helix I to IV and VI-IX and selected 51 residues as putative substrate binding sites based on solvent accessibility (**Figure 1**). We hypothesised that the transport cavity contains highly conserved residues that are key for the transport activity. To test this, we aligned 97 protein sequences consisting of homologs of UMAMIT clade I from 27 plant species and created sequence logos containing the 51 residues identified in the structural analysis (**Supplementary Figure 1**). Four residues within the binding cavity—R44, G82, W200, and Q204—are 100% conserved among all the sequences in the multiple sequence alignment (**Supplementary Figure S2A**). This suggests that these residues are crucial for functional or structural features of the transporter proteins. When mutating, respectively, R44, W200, and Q204 into alanine in UMAMIT29 (UMAMIT29#3CON), the import activity of aliphatic 4-methylthiobutyl glucosinolate (4MTB) and benzyl glucosinolate (BGLS) was reduced by over 80%, and indol-3-ylmethyl glucosinolate (I3M) was reduced by approximately 50% (**Supplementary Figures S2B–D**). Interestingly, altering only R44 (UMAMIT29#R44A) reduced all tested glucosinolate transport activity by 88%–98% (**Supplementary Figures S2B–D**). The identification of essential residues for glucosinolate transport activity within the predicted substrate transporting cavity supports our model.

### 2.2 Key residues for glucosinolate transport activity of UMAMIT29

**Figure 2:**
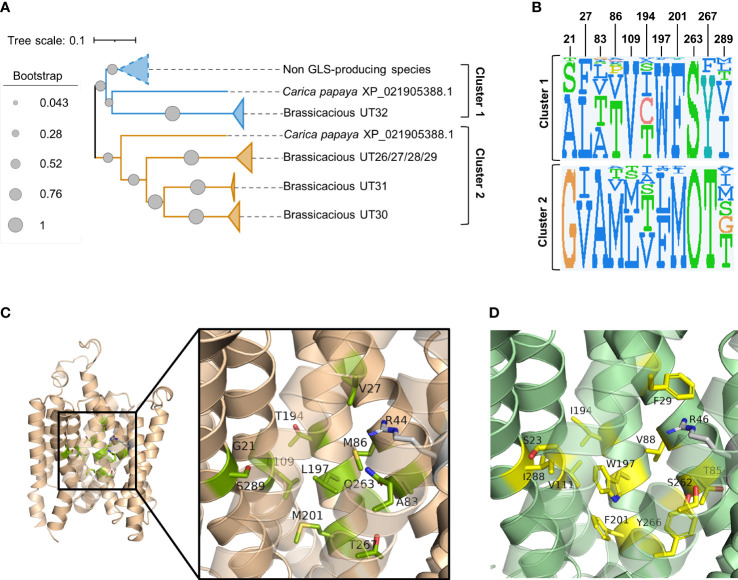
Prediction of glucosinolate-binding residues through analysis of differentially conserved amino acid residues in UMAMIT29 and UMAMIT32 homologs. **(A)** Schematic phylogenetic tree of UMAMITs (derived from the phylogenetic tree displayed in (**Supplementary Figure S2**) reveals two major clusters comprising the non-brassicaceous-specific UMAMIT32 homologs (cluster 1) and the brassicaceous-specific UMAMIT29–31 homologs (cluster 2). **(B)** Sequence logos of 11 differentially conserved residues in the binding pocket of UMAMIT29 and UMAMIT32 made from the sequences from homologs in clusters 1 and 2. The sequence logos were made in JDet (“O” represents the one letter code “Q” for glutamine by the programme). The numbers constitute the residue position in UMAMIT29 (as shown in **Supplementary Table S1**). The sequence logo of all 51 residues within the predicted substrate transporting cavity can be seen in **Supplementary Figure S3**. **(C)** Protein model of UMAMIT29 (orange) depicting the 11 predicted differentially conserved residues in UMAMIT29 (light green). **(D)** Protein model of UMAMIT32 (dark green) depicting the 11 predicted differentially conserved residues in UMAMIT32 (yellow).

Phylogenetic analysis of the above 97 sequences shows that the homologs of brassicaceous-specific, glucosinolate-transporting UMAMITs and non-brassicaceous-specific, non-glucosinolate-transporting UMAMITs fall into two different clusters (**Figure 2A**; **Supplementary Figure S1**). We hypothesised that differentially conserved residues located in the binding cavity of the proteins within the two clusters may reveal specificity-determining positions.

Analysis of the sequence logos of the 51 predicted substrate-transporting cavity residues between the two clusters identified 13 differentially conserved residues (**Supplementary Figure S3**; **Supplementary Table S1**). Two amino acid residues among the 13 positions were identical between the non-glucosinolate-transporting UMAMIT32 and the glucosinolate-transporting UMAMIT30 and UMAMIT31 and were therefore not considered further (**Figure 2B**; **Supplementary Figure S3**).

**Figure 3:**
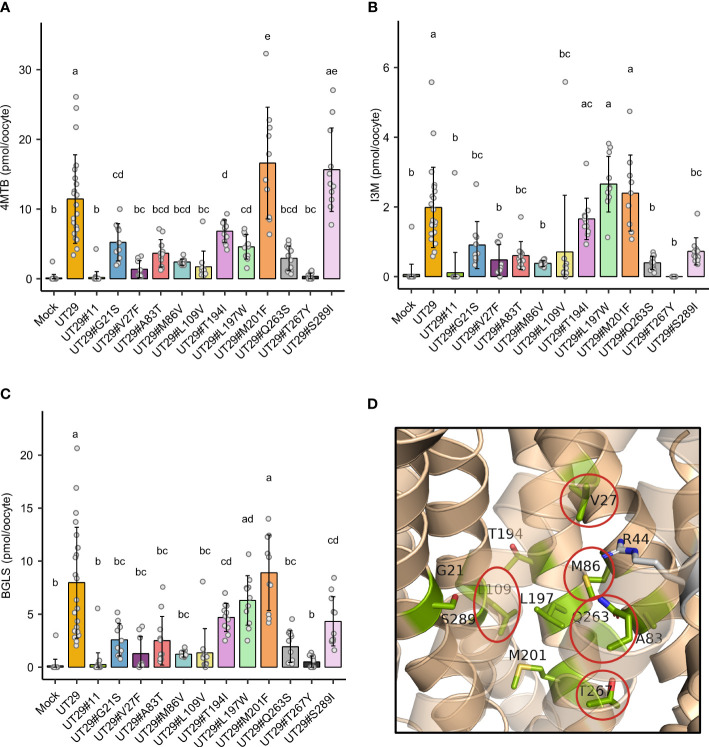
Glucosinolate import activity of 11 mutant variants of UMAMIT29. **(A–C)** Import of 4MTB, I3M, and BGLS by mutant variants of UMAMIT29 (equimolar concentration of 200 µM of each glucosinolate in the buffer) at pH5.5 in *Xenopus* oocytes. Bar plots show intracellular levels of 4MTB **(A)**, I3M **(B)** and BGLS **(C)** after 1 h incubation. The different lowercase letters above each bar in the chart indicate significant differences in the mean (one-way ANOVA followed by Tukey HSD test, p ≤ 0.05). Data were collected from at least two independent experiments. **(D)** Protein model of UMAMIT29 (orange) marking the five key residues (red circle) among the 11 differentially conserved residues (light green). UT, UMAMIT; 4MTB, 4-methylthiobutyl glucosinolate; I3M, indole 3-ylmethyl glucosinolate; BGLS, benzyl glucosinolate.

Subsequently, we set out to examine the contributions of the 11 differentially conserved residues to glucosinolate transport activity by generating 11 UMAMIT29 mutant variants in which each of the 11 residues was individually changed into the corresponding residue from UMAMIT32 (**Figures 2C, D**). The glucosinolate substrate specificity of the mutant variants were compared to wildtype UMAMIT29 that shows broad substrate specificity towards methionine-derived aliphatic glucosinolates and tryptophan-derived indolic glucosinolates (Xu et al., 2023). The mutant variants were expressed in *Xenopus laevis* oocytes and tested for import of a mixture of three glucosinolates, the aliphatic 4-methylthiobutyl glucosinolate (4MTB), indol-3-ylmethyl glucosinolate (I3M), and benzyl glucosinolate (BGLS). Eight of the 11 mutant variants showed reduced transport activity to all three glucosinolates. Among them, UMAMIT29#V27F, UMAMIT29#M86V, UMAMIT29#L109V, UMAMIT29#Q263S, and UMAMIT29#T267Y showed the most reduced activity with 75%–97% less total glucosinolate import compared to the wild-type UMAMIT29 upon expression in the oocytes (**Figures 3A–C**; **Table 1**). Although all these mutant variants have big reduction in total glucosinolate import activity, four of them - UMAMIT29#M86V, -L109V, -Q263S and -T267Y - locate in near proximity to the centre of the cavity, whereas UMAMIT29#V27F locates further distally at the end of helix I in the computed protein model (**Figure 3D**). UMAMIT29#L197W showed a reduction in 4MTB import, compared to the wild type, but the level of imported I3M and BGLS was not affected.. UMAMIT29#S289I showed reduction in I3M and BGLS import and the level of imported 4MTB was similar to wild type (**Figures 3A–C**; **Table 1**). UMAMIT29#M201F showed—as the only mutant variant—no reduction in any glucosinolates, but an increase in 4MTB (**Figures 3A–C**; **Table 1**).

**Table 1:** Glucosinolate import activity of mutant variants of UMAMIT29.

Protein	4MTB		I3M		BGLS		Sum of GLS	
	pmol^1^	%^2^	pmol^1^	%^2^	pmol^1^	%^2^	pmol^1^	%^2^
Mock	0.11 ± 0.52		0.06 ± 0.30		0.13 ± 0.63		0.30 ± 1.45	
UT29	11.44 ± 6.36		1.99 ± 1.15		7.97 ± 5.22		21.39 ± 12.16	
UT29#11	0.18 ± 0.84	98↓	0.11 ± 0.58	94↓	0.26 ± 1.09	97↓	0.56 ± 2.51	97↓
UT29#G21S	**5.23 ± 2.66**	**54↓**	**0.91 ± 0.68**	**54↓**	**2.58 ± 1.53**	**68↓**	8.72 ± 4.64	59↓
UT29#V27F	**1.38 ± 1.21**	**88↓**	**0.48 ± 0.44**	**76↓**	**1.29 ± 1.60**	**84↓**	3.15 ± 3.21	85↓
UT29#A83T	**3.65 ± 1.97**	**68↓**	**0.61 ± 0.40**	**69↓**	**2.50 ± 2.30**	**69↓**	6.76 ± 4.54	68↓
UT29#M86V	**2.39 ± 2.23**	**79↓**	**0.38 ± 0.09**	**81↓**	**1.24 ± 0.29**	**84↓**	4.01 ± 0.74	81↓
UT29# L109V	**1.74 ± 2.23**	**85↓**	**0.71 ± 1.62**	**64↓**	**1.36 ± 2.29**	**83↓**	3.81 ± 6.13	82↓
UT29#T194I	**6.83 ± 1.60**	**40↓**	1.66 ± 0.60	17↓	**4.70 ± 1.33**	**41↓**	13.19 ± 3.31	38↓
UT29#L197W	**4.58 ± 1.75**	**60↓**	2.65 ± 0.80	34↑	6.29 ± 2.33	21↓	13.52 ± 4.75	37↓
UT29#M201F	**16.59 ± 8.01**	**45↑**	2.40 ± 1.09	21↑	8.91 ± 3.56	12↑	27.90 ± 12.54	30↑
UT29#Q263S	**2.93 ± 1.78**	**74↓**	**0.40 ± 0.19**	**80↓**	**1.94 ± 1.50**	**76↓**	5.27 ± 3.43	75↓
UT29#T267Y	**0.36 ± 0.44**	**97↓**	**0.00 ± 0.00**	**100↓**	**0.51 ± 0.56**	**94↓**	0.87 ± 0.95	96↓
UT29#S289I	15.66 ± 6.00	37↑	**0.73 ± 0.39**	**63↓**	**4.32 ± 2.32**	**46↓**	20.71 ± 8.42	3↓

^1^Mean ± SD.

^2^% higher (↑) or lower (↓) trend of import activity compared to wild type UMAMIT29.

Mutant variants were expressed in *Xenopus laevis* oocytes, and import activity was measured over 60 min. The percentage activity is given relative to wild type UMAMIT29. For each datapoint, three oocytes were pooled as one biological replicate, and data from four to five biological replicates are shown. The values marked in bold indicate significant differences in glucosinolate levels between the mutant variant and wild type UT29 (one-way ANOVA followed by Tukey HSD test, p ≤ 0.05).

UT, UMAMIT; GLS, glucosinolates; 4MTB, 4-methylthiobutyl glucosinolate; I3M, indole 3-ylmethyl glucosinolate; BGLS, benzyl glucosinolate.

## 3 Discussion

In this study, we have identified key amino acid residues within the predicted substrate-transporting cavity of glucosinolate-transporting UMAMIT29 by comparing to non-glucosinolate-transporting UMAMIT32. When 11 differently conserved amino acid residues were tested for their role in determining glucosinolate transport activity by substitution of each of those from UMAMIT29 with the corresponding residues in UMAMIT32, five mutant variants caused over 75% reduction in total glucosinolate import activity (**Figures 3A–D**; **Table 1**). As we only exchange a single amino acid residue in UMAMIT29 with the corresponding amino acid residue in the homolog UMAMIT32, we anticipate that the UMAMIT29 mutant variants are being functionally expressed. This is supported by the observation that variant UMAMIT29#S289I shows specific reduction in the import of the I3M and BGLS and that variant UMAMIT29#L197W imports less 4MTB but similar levels of I3M and BGLS compared to wild type. The alteration of glucosinolate substrate specificity of these UMAMIT29 mutant variants suggests that these residues are critical for glucosinolate binding.

Noticeably, amongst the substitution of the five single residues of UMAMIT29 with the markedly lowered glucosinolate transport activity, UMAMIT29#V27F , UMAMI T 2 9#M86V, UMAMIT29#L109V, and UMAMIT29# Q263S mutant variants do not change hydrophobicity properties of original amino acids, whereas the UMAMIT29#T267Y mutant variant exchanges a polar residue to a larger and hydrophobic residue. We do, however, not observe a clear spatially distinguished subdomain for the five key residues compared with the remaining six residues. This is different from the glucosinolate importer NPF2.9 and NPF2.10 of which the key residues involved in glucosinolate selectivity form a ring structure (Kanstrup et al., 2023). This suggests that the identified key residues within the cavity in UMAMIT29 play a role in different steps of the transport cycle. Consistently, the five key residues are not located in helix 5 or 10, which are proposed to be essential for dimerisation, which is required for transport of amino acids in UMAMIT14 (Zhao et al., 2021). This suggests that these residues are key for UMAMIT29-mediated glucosinolate transport activity and thus potential targets for blocking the transport of glucosinolates to the seeds. Additionally, identification of key residues determining glucosinolate transport may provide insights into the determinants of substrate specificity of UMAMIT transporters and pave the way to understanding the evolution of transporter specificity from primary metabolites to specialized metabolites within the UMAMIT family.

## 4 Materials and methods

### 4.1 Mining amino acid sequences of UMAMIT homologs

The amino acid sequences of UMAMIT clade I (UMAMIT26–32) were obtained from TAIR (https://www.arabidopsis.org) for *A. thaliana*, BnPIR (http://cbi.hzau.edu.cn/bnapus/index.php) (Song et al., 2020) for *Brassica napus* ZS11 and Genoscope ((Belser et al., 2018), https://www.genoscope.cns.fr/externe/plants/index.html) for *Brassica rapa Z1* and *Brassica oleracea HDEM*, phytozome 13 (https://phytozome-next.jgi.doe.gov) for *Manihot esculenta*, and NCBI for *Physcomitrella patens.* Sequences of the other plant species were adopted from Zhao et al. (2021), except for MaCap/01 (NCBI ID: XP_023632698.1), MaCar/15 (NCBI ID: XP_021905388.1), and MaCar/16 (NCBI ID: XP_021887563.1), which were retrieved from NCBI.

### 4.2 Multiple sequence alignment and phylogenetic analysis

Sequences from 27 plant species were aligned in MEGA X (https://www.megasoftware.net/)(Kumar et al., 2018) using MUSCLE with default settings (Edgar, 2004). Sequences with indels within any helices were removed, with the exceptions of AsSol/42, FaMed/43 and the root. The final alignment contained 97 sequences. Sequence logos were made using JDet (http://csbg.cnb.csic.es/JDet/) (Muth et al., 2012). Phylogenetic trees were generated in MEGAX using the neighbour-joining method (1,000 bootstraps) and annotated in iTOL (Letunic and Bork, 2021).

### 4.3 Estimation of differentially conserved amino acids using Diverge 3.0 beta 1

DIVERGE 3.0 beta 1 was used to determine differentially conserved amino acids (https://github.com/xungulab/diverge) (Gu et al., 2013). The multiple sequence alignment with 97 sequences and its corresponding neighbour-joining phylogenetic tree were loaded into the program. The estimation of cluster-specific functional divergence (corresponding to the amount of differential conservation), was calculated based on the algorithm in Gu et al. (2013), and the final scores are listed in the **Supplementary Table S1**.

### 4.4 Protein modelling and analysis

Structures of the *Arabidopsis* UMAMIT29 (Uniprot ID: Q9M131) and *Arabidopsis* UMAMIT32 (Uniprot ID: Q9LI65) were modelled using RaptorX from (http://raptorx.uchicago.edu/ContactMap/)(Ma et al., 2015; Wang et al., 2016; Wang et al., 2017) and AlphaFold (https://alphafold.ebi.ac.uk/)(Senior et al., 2020; Jumper et al., 2021). Protein models were depicted by The PyMOL Molecular Graphics System, Version 2.4 Schrödinger, LLC (https://pymol.org/2/). The residues constituting the active site of UMAMIT29 and UMAMIT32, respectively, were determined using the CAVER webtool v1.0 (https://loschmidt.chemi.muni.cz/caverweb/) (Stourac et al., 2019) followed by manual inspection and minor revisions in PyMOL 2.4.

### 4.5 Generation of UMAMIT29 mutant variants

DNA fragments of UT29#11, i.e., UMAMIT29 with the 11 differentially conserved residues from UMAMIT32, and UT29#3CON, i.e., the UMAMIT29 with the three UMAMIT-conserved residues mutated into alanine, were ordered from Twist Bioscience. UMAMIT29 single residue mutant variants were generated through USER cloning (Nour-Eldin et al., 2006). Linear DNA template for *in vitro* transcription was obtained from PCR amplification of the pNB1u plasmids using Phusion High-Fidelity DNA polymerase (NEB) and PCR product purified using the E.Z.N.A^®^ Cycle Pure Kit (Omega Bio-tek). Template DNA was *in vitro* transcribed using the mMessage mMachine™ T7 transcription kit (InVitrogen). The RNA transcripts (600 ng/µl) were aliquoted into 10 μl per tube and kept at −18°C until use.

### 4.6 Measurement of transport activity in *Xenopus* oocytes

Transport assays using *Xenopus* oocytes were described in Xu et al. (2016). Briefly, the defolliculated *Xenopus laevis* oocytes (stage V or VI) were ordered from Ecocyte Bioscience or Department of Drug Design and Pharmacology, University of Copenhagen. Oocytes were injected with 50 nl RNA (600 ng/µl) using a Nanoject II (Drummond Scientific Company). For the mock (negative control), oocytes were injected with 50 nl sterilised Milli-Q^®^ H_2_O. The injected oocytes were used for assaying after 3 days of incubation at 16°C in Kulori buffer pH 7.4 (5 mM MES, 90 mM NaCl, 1 mM KCl, 1 mM CaCl_2_, and 1 mM MgCl_2_) supplemented with gentamicin (100 µg/ml).

The assays were performed as follows: Oocytes were first pre-incubated in Kulori buffer pH 5 (5 mM MES, 90 mM NaCl, 1 mM KCl, 1 mM CaCl_2_, and 1 mM MgCl_2_) without substrates for 5 min. Then, oocytes were incubated for 1 h in Kulori buffer pH 5 (5 mM MES, 90 mM NaCl, 1 mM KCl, 1 mM CaCl_2_, and 1 mM MgCl_2_) with added 4-methylthiobutyl glucosinolate (4MTB), indol-3-ylmethyl glucosinolate (I3M), and benzyl glucosinolate (BGLS) (200 µM of each). After 1 h, oocytes were washed in five Petri dishes containing Milli-Q^®^ H_2_O. From the final Petri dish, oocytes were divided into Eppendorf tubes^®^ with three oocytes in each. Residual H_2_O was removed from each of the tubes and the oocytes homogenized in 50 μl 50% methanol containing an internal standard of 1.25 µM sinigrin (62.5 pmol per sample). The tubes were placed at −18°C overnight.

### 4.7 Sample preparation for LC/MS analysis

Glucosinolates were extracted and quantified as desulfo-glucosinolates as previously described (Jensen et al., 2015; Crocoll et al., 2016). Briefly, a 96-well filter plate (0.45 µM) was filled with 45 µl DEAE-Sephadex A-25 using a MultiScreen Column Loader (Merck Millipore). A total of 300 µl H_2_O was added to each well, and the plate was incubated 3–4 h at room temperature or overnight in the fridge. Excess H_2_O was removed by applying 2–4 s of vacuum using a vacuum manifold. After centrifugation for >15 min at >20,000 × *g* at 4°C, all supernatant from the oocyte extraction was added to the DEAE-Sephadex, and vacuum was applied for 2–4 s. The wells were then washed two times with 100 µl 70% methanol and two times with 100 µl H_2_O using 2–4 s of vacuum. In the final washing step, the plate was spun for <15 s at 5,900 rpm. Sulphatase (20 µl) was added to each well, and the plate was incubated overnight at room temperature. Desulfo-glucosinolates were eluted with 90 µl of H_2_O at 5,900 rpm. The plate was kept at −18°C until LC/MS analysis.

### 4.8 Data analysis and statistics

Plots and statistics were made with R studio (version 2021.09.0 + 351). Statistics was done by first performing a one-way ANOVA test of the compound to the RNA followed by a Tukey honest significant differences (Tukey HSD) test for a multiple pairwise comparison of the mean for each of the RNAs (p<0.05).

## Data availability statement

Source data presented in this study are provided in the article/Supplementary material/ Table 1.xlsx.

## Author contributions

DX, LM, and BH contributed to the conception and design of the study. LM performed all the experiments, including bioinformatics analysis, characterisation of transporters, and data analysis, supervised by DX, OM, and BH. CC performed LC–MS/MS analyses. DX, LM, OM, and BH wrote the article based on a draft provided by LM. All authors contributed to the article and approved the submitted version.
