# Supplementary material for: Identification of key amino acid residues in AtUMAMIT29 for transport of glucosinolates
Source: Front Plant Sci. 2023 Jul 17;14:1219783. doi: 10.3389/fpls.2023.1219783 (PMC10388549; doi:10.3389/fpls.2023.1219783)
Supplement: Supplementary file 2 [file Presentation_1.pdf]

Supplementary Material

Identification of key amino acid residues in AtUMAMIT29 for transport of glucosinolates

Lasse Meyer[1], Christoph Crocoll[1], Barbara Ann Halkier[1], Osman Asghar Mirza[2*], Deyang Xu[1*]

*** Correspondence: Deyang Xu**: dyxu@plen.ku.dk

Co-corresponding author: Osman Asghar Mirza om@sund.ku.dk

1 Supplementary data

The source data for the study has been uploaded as an independent file “Meyer et al source data.xlsx”

2 Supplementary Figures and Tables

2.1 Supplementary Figures

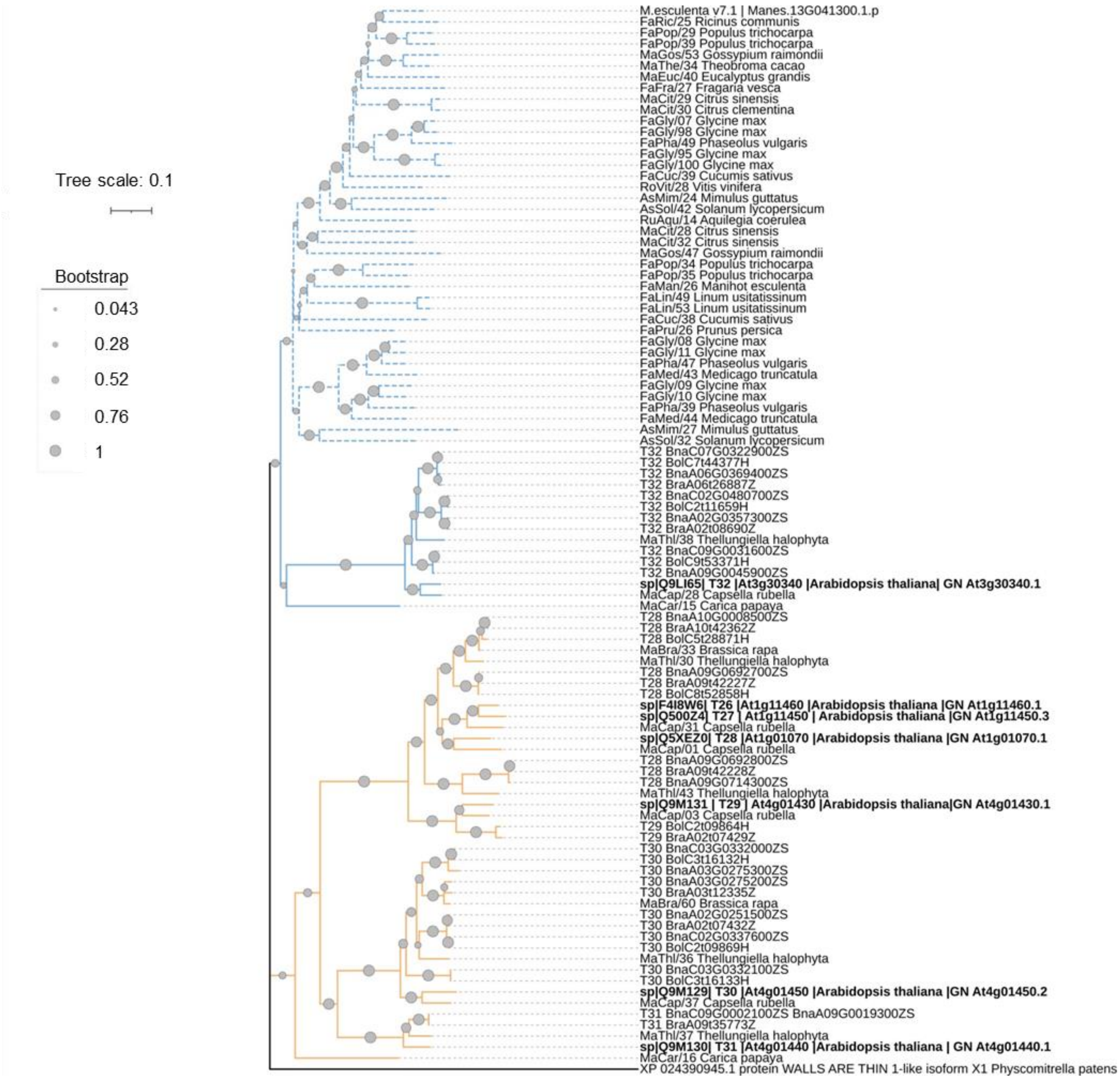

Supplementary Figure 1. Phylogenetic tree of the UMAMIT Clade I from 27 species. Brassicaceous-specific homologs (orange) and non-brassicaceous-specific homologs (blue) of UMAMIT clade I proteins form two major subclades. UMAMIT26-UMAMIT32 sequences from *Arabidopsis thaliana* are shown in bold. The phylogenetic tree was generated in MEGAX using the neighbour-joining method (1000 bootstraps) and annotated in iTOL.

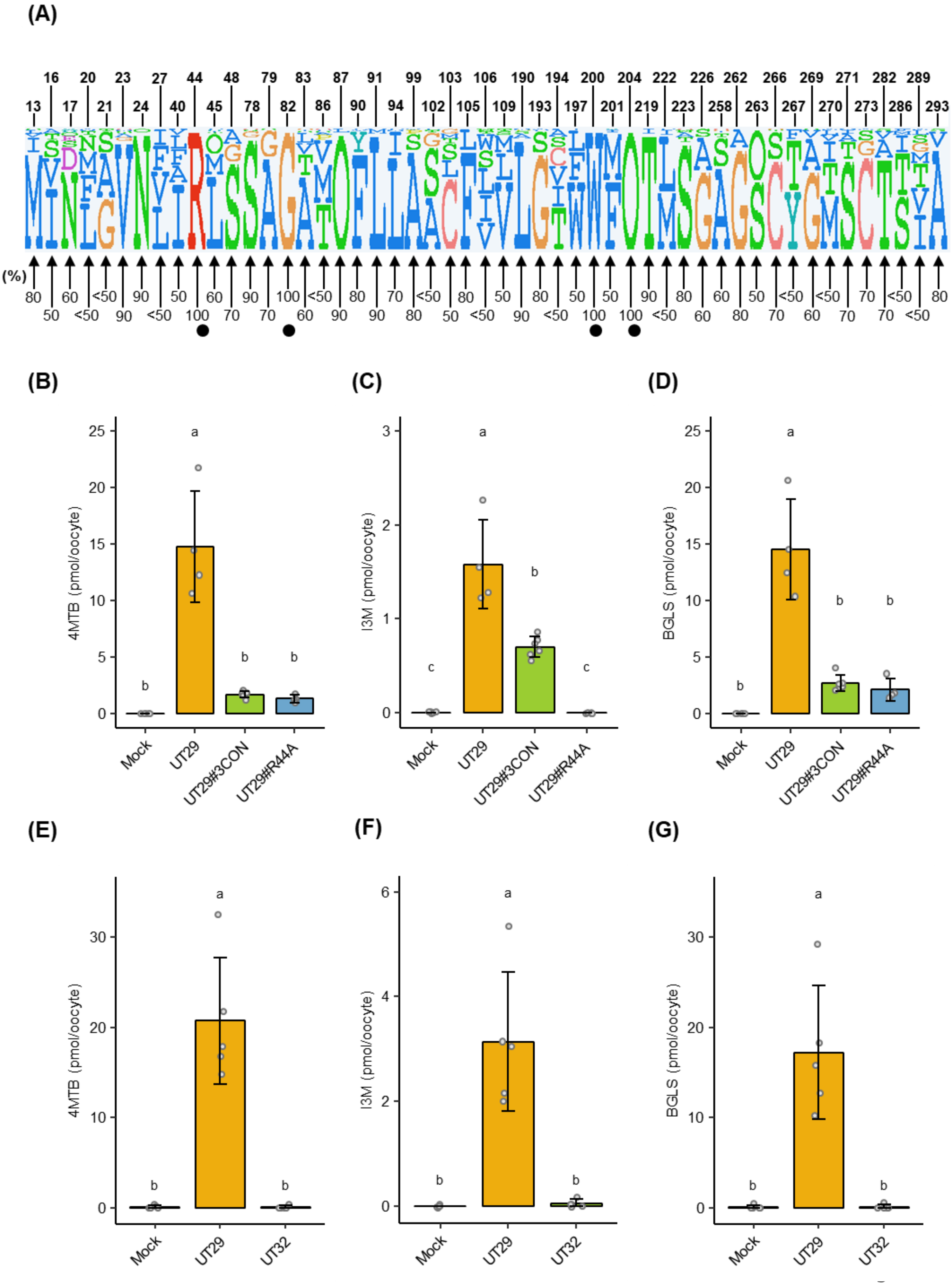

(A)
(%)
(B)
(C)
(D)
4MTB (pmol/oocyte)
I3M (pmol/oocyte)
BGLS (pmol/oocyte)
Mock
UT29
UT29#3CON
UT29#R44A
(E)
(F)
(G)
UT32

Supplementary Figure 2: Glucosinolate import activity of the UMAMIT29 mutant variants mutated in conserved residues within the predicted substrate transporting cavity. (**A**) Sequence logo of the 51 residues predicted to constitute substrate transporting cavity based on amino acid sequences of 96 transporters of the UMAMIT clade I. The % conservation of each residue related to all 96 sequences is shown. Four of the amino acid residues are 100% conserved and marked with black dots. The sequence logo was made in JDet ("O" represents the one letter code "Q" for glutamine by the program). The numbers constitute the residue position in UMAMIT29 (as shown in Supplementary Table S1) (**B-G**) Import of 4MTB, I3M and BGLS by mutant variants of UMAMIT29 (**B-D**) and UMAMIT32 (**E-G**) (equimolar concentrations of 200 µM of each glucosinolate in the buffer) at pH 5 in Xenopus oocytes. The different lowercase letters above each bars indicate significant differences of the mean (one-way ANOVA followed by TUKEY HSD test, $p < 0.05$). Abbreviations: UT, UMAMIT; 4MTB, 4-methylthiobutyl glucosinolate; I3M, indole 3-ylmethyl glucosinolate; BGLS, benzyl glucosinolate.

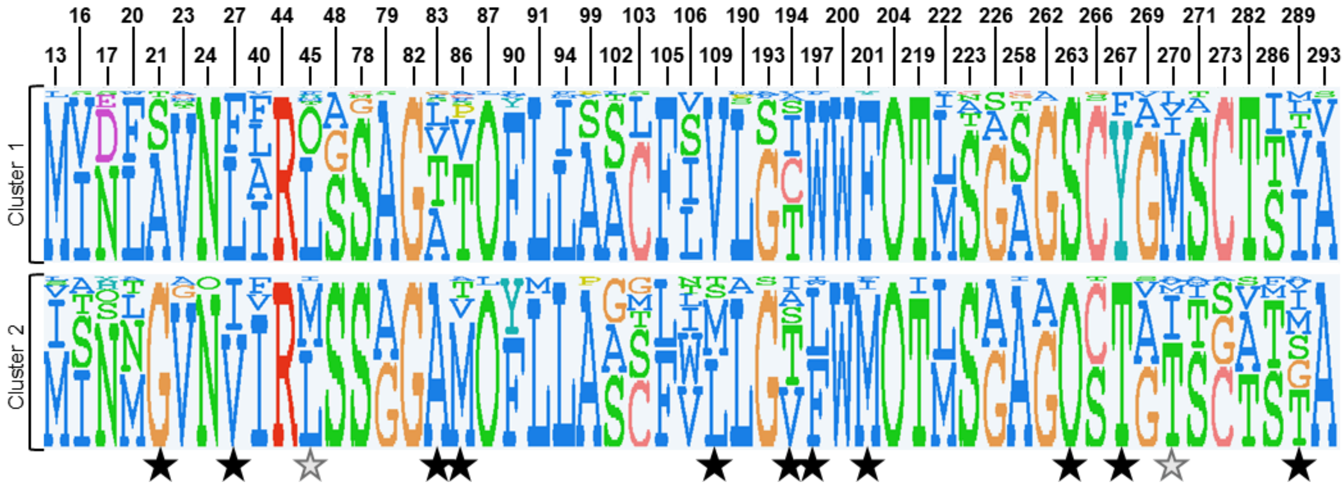

Supplementary Figure 3: Sequence logos of amino acid residues in the predicted substrate transporting cavities of the two major clusters of transporters of UMAMIT clade I. Differentially conserved residues (stars) were estimated using the DIVERGE software. Two of the estimated residues from the DIVERGE estimation were identical among UMAMIT32 and respectively, UMAMIT30 and UMAMIT31 and therefore filtered out (grey stars). The resulting 11 residues estimated to be differentially conserved were tested experimentally for their role in glucosinolate transport activity (black stars). The sequence logos were made in JDet ("O" represents the one letter code "Q" for glutamine by the program). The numbers constitute the residue position in UMAMIT29 (as shown in Supplementary Table S1).

2.2 Supplementary Table

Supplementary table 1: The amino acid residues predicted to constitute the substrate transporting cavity of *Arabidopsis* UMAMIT29 and UMAMIT32.

**Residue position in UT29**	**UT29 amino acid residue**	**UT30 amino acid residue**	**UT31 amino acid residue**	**UT32 amino acid residue**	**Diverge score**
13	M	L	M	M	0.000
16	S	S	I	I	0.481*
17	S	N	N	N	0.000
20	M	A	L	L	0.000
**21**	**G**	**G**	**G**	**S**	**2.910**
23	V	V	A	V	0.000
24	N	N	N	N	0.000
**27**	**V**	**V**	**V**	**F**	**1.184**
40	F	I	I	A	0.000
44	R	R	R	R	0.000
45	M	L	L	L	1.398*
48	S	S	S	G	0.000
78	S	S	S	S	0.000
79	G	A	A	A	0.000
82	G	G	G	G	0.000
**83**	**A**	**A**	**A**	**T**	**0.585**
**86**	**M**	**M**	**T**	**V**	**1.263**
87	Q	Q	Q	Q	0.000
90	F	Y	F	F	0.000
91	L	L	L	L	0.000
94	L	L	L	L	0.000
99	A	A	A	S	0.000
102	S	G	A	S	0.000
103	M	S	C	L	0.000
105	L	F	F	F	0.000
106	V	W	I	S	0.000
**109**	**L**	**M**	**T**	**V**	**1.468**
190	L	L	L	L	0.000
193	G	G	G	S	0.000
**194**	**T**	**V**	**S**	**I**	**0.524**
**197**	**L**	**F**	**F**	**W**	**1.300**
200	W	W	W	W	0.000
**201**	**M**	**M**	**M**	**F**	**2.020**
204	Q	Q	Q	Q	0.000
219	T	T	T	T	0.000
222	M	L	L	L	0.838*
223	S	S	S	S	0.000
226	A	G	G	G	0.000
258	A	A	A	S	NA
262	G	A	A	G	0.000
**263**	**Q**	**Q**	**Q**	**S**	**7.592**
266	S	C	C	C	0.000
**267**	**T**	**T**	**T**	**Y**	**2.444**
269	V	G	G	G	0.000
270	T	M	T	M	1.191*
271	S	S	S	S	0.000
273	S	C	C	C	0.000

282	V	S	T	T	0.000
286	S	S	T	I	0.000
**289**	**S**	**V**	**G**	**I**	**0.707**
293	A	A	A	A	0.000

Residues in bold indicate each of the 11 differentially conserved amino acids identified in the *in silico* analyses. NA: Not available. * residues with a score ≠0 that has identical residues among UT30/UT31 and UT32. Abbreviations: UT, UMAMIT.
